# Preclinical evidence of the therapeutic effect of *Moringa oleifera* in peptic ulcer disease: a systematic review and meta-analysis

**DOI:** 10.3389/fphar.2026.1689789

**Published:** 2026-03-20

**Authors:** Saheed Olatunbosun Akiode, Adeoti Gbemisola Adeniran, Oyedayo Phillips Akano, Ayokunmi Stephen Olusa, Oghale Oghenefego Ovuakporie-Uvo, Oluchukwu Perpetual Okeke, Olunike Rebecca Abodunrin, Folahanmi Tomiwa Akinsolu, Uwaisu Iliyasu, Olajide Odunayo Sobande, Kamoru Abdulazeez Adeniyi

**Affiliations:** 1 Biotechnology Advanced Research Centre, Sheda Science and Technology Complex (SHESTCO), Garki, Nigeria; 2 Department of Physiology, University of Medical Sciences, Ondo City, Nigeria; 3 Department of Physiology, Faculty of Basic Medical Sciences, Olabisi Onabanjo University, Ago Iwoye, Ogun, Nigeria; 4 Department of Pharmacology, Faculty of Pharmacy, Obafemi Awolowo University, Ile-Ife, Osun State, Nigeria; 5 Department of Science Laboratory Technology/Centre for Herbal Medicine and Drug Discovery, University of Medical Sciences, Ondo City, Ondo State, Nigeria; 6 Nigerian Institute of Medical Research Foundation, Yaba, Lagos State, Nigeria; 7 Department of Epidemiology and Biostatics, Nanjing Medical University, Nanjing, China; 8 Center for Reproduction and Population Studies, Clinical Sciences Department, Nigerian Institute of Medical Research, Yaba, Lagos State, Nigeria; 9 Department of Research, Capacity Building and Implementation, Alabastron Initiative, Lagos, Nigeria; 10 Department of Public Health, Faculty of Basic Medical and Health Sciences, Lead City University, Ibadan, Nigeria; 11 Department of Pharmacognosy and Drug Development, Kaduna State University, Kaduna, Nigeria; 12 Department of Animal and Environmental Biology, Federal University Dutse, Dutse, Jigawa State, Nigeria

**Keywords:** anti-ulcer, gastric ulcer, gastroprotective, meta-analysis, *Moringa oleifera*, peptic ulcer disease, systematic review, ulcer index

## Abstract

**Background:**

Peptic ulcer disease (PUD) remains a global health burden, with current therapies limited by recurrence, adverse effects, and high costs. *Moringa oleifera*, a medicinal plant traditionally used for gastrointestinal disorders, has shown anti-ulcer potential in several experimental models. However, no systematic synthesis has consolidated the available preclinical evidence. This review evaluated the therapeutic efficacy of *M. oleifera* in PUD using evidence from preclinical (rodent) studies.

**Materials and Methods:**

We searched PubMed, CINAHL, Scopus, Web of Science, and the Cochrane Library from inception (without time filter) to August 2025. Eligible studies were randomized controlled animal experiments assessing *M. oleifera* extracts in ulcer-induced models. Data on ulcer index were extracted, and a random-effects meta-analysis was performed using Hedge’s g to calculate standard mean difference (SMD). Heterogeneity was assessed with I^2^, and publication bias with Egger’s regression and trim-and-fill methods.

**Results:**

Eleven (11) studies involving 268 animals met the inclusion criteria, with SYRCLE quality scores ranging from 8 to 9. Pooled analysis showed that *M. oleifera* significantly reduced ulcer index compared with controls (SMD = −1.42; 95% CI: −2.01 to −0.83; p < 0.01). Subgroup analyses revealed variability by dose and comparator. High-dose comparisons with standard drugs showed no significant difference (SMD = −0.03; 95% CI: −0.27 to 0.66; p = 0.92; I^2^ = 69%), while comparisons with basal controls also lacked significance (SMD = 0.99; 95% CI: 0.24–2.22; p = 0.11). Sensitivity analyses resolved heterogeneity (I^2^ = 0%) and publication bias, without altering overall outcomes.

**Conclusion:**

*Moringa oleifera* demonstrates consistent gastroprotective and ulcer-healing effects in preclinical studies, though not superior to standard therapies. Methodological heterogeneity and the absence of clinical trials highlight the need for standardized experimental protocols and translational research to establish its role as a potential adjunctive therapy for PUD.

**Systematic Review Registration:**

Identifier CRD420251080346.

## 1 Introduction

Peptic ulcer disease (PUD) is a common gastrointestinal disorder characterized by disruption of the gastric or duodenal mucosa, often accompanied by acute or chronic inflammation (Devaraj et al., 2007). Major etiological factors include the mucosal injury induced by nonsteroidal anti-inflammatory drugs (NSAIDs), ethanol, and stress, which are widely used in experimental ulcer models (Dalhoumi et al., 2022; Ibrahim and Al-Qadhi, 2025; Ahmed et al., 2021).

Lifestyle factors such as alcohol use and smoking further exacerbate disease progression. Although proton pump inhibitors (PPIs) and H2-receptor antagonists remain the mainstay of therapy, their limitations—including adverse effects, recurrence, and high cost—have prompted growing interest in alternative or adjunctive approaches (Dalhoumi et al., 2022; Ibrahim and Al-Qadhi, 2025). In resource-limited settings, plant-based therapies are particularly attractive due to their affordability, accessibility, and established ethnomedicinal use.

*Moringa oleifera* Lam (Moringaceae), commonly known as the drumstick tree, is native to Africa and Asia and has been widely applied in traditional medicine, including for gastrointestinal disorders (Ahmed et al., 2021; Pareek et al., 2023). Nearly all parts of the plant, leaves, bark, roots, seeds, and flowers, contain bioactive compounds with reported pharmacological activities. Preclinical studies have demonstrated that *M. oleifera* extracts can reduce ulcer incidence by lowering gastric acid secretion, enhancing mucus production, and mitigating oxidative stress (Devaraj et al., 2007; Alissa et al., 2025; Choudhary et al., 2013; Lawal et al., 2018; Wahba and Shelbaya, 2018). Both aqueous and methanolic extracts have shown preventive and curative effects in animal models, though clinical validation remains limited (Dalhoumi et al., 2022; El Mahdy et al., 2020).

The therapeutic potential of *M. oleifera* is supported by its rich phytochemical profile, including quercetin, kaempferol, chlorogenic acid, and isothiocyanates, which modulate inflammatory mediators, inhibit lipid peroxidation, and restore mucosal integrity (Jo et al., 2020; Serafim et al., 2020; Fahey, 2017). Flavonoids such as quercetin stabilize mast cells and reduce histamine secretion, while isothiocyanates promote nitric oxide synthase activity, enhancing vasodilation and mucosal repair (Pareek et al., 2023; Fahey, 2017). However, these findings remain fragmented across studies with differing ulcer-induction models, extraction methods, dosages, and outcome measures. This heterogeneity hampers cross-study comparisons and obscures consensus on efficacy and safety. Moreover, data on long-term toxicity, herb–drug interactions, and reproducibility are scarce, and translation from animal models to human application remains uncertain.

Herbal medicines have been reported to possess several advantages when compared to synthetic drugs in the management of PUD. They are often cost-effective, readily available, and associated with fewer side effects than other drugs. Phytochemicals, such as flavonoids, alkaloids, tannins, and saponins, found in ethnomedicinal plants like *Moringa oleifera*, *Azadirachta indica*, *Glycyrrhiza glabra*, and *Curcuma longa*, exhibit anti-*Helicobacter pylori*, anti-inflammatory, and gastroprotective properties (Choudhary et al., 2013; Egbe et al., 2025).

*Moringa oleifera* is often considered preferable to other ethnobotanical plants in PUD management due to its multifaceted therapeutic profile and comparatively well-documented pharmacological safety (Dalhoumi et al., 2022; Soto et al., 2025). Specifically, the five major protective actions combined in *M. oleifera*: cytoprotection, anti-inflammatory activity, antioxidant effect, antimicrobial effect (anti-*H. pylori*), and nutritional safety, which is not obtainable in other ethnobotanical plants, informed the choice of *M. oleifera* in this study.

Despite the growing number of experimental studies, no systematic review or meta-analysis has consolidated preclinical evidence on *M. oleifera* for PUD. Such synthesis is crucial to clarify its pharmacological mechanisms, assess the consistency of findings, and guide the rational design of clinical trials, particularly in low-resource contexts where cost-effective therapies are needed.

Accordingly, this systematic review and meta-analysis aimed to evaluate the therapeutic effects of *M. oleifera* in preclinical models of PUD. Specifically, it sought to (1) assess the extent of preclinical and clinical evidence supporting its use; (2) examine pharmacological mechanisms underlying its gastroprotective effects; (3) compare the efficacy of different plant parts and extraction methods; and (4) evaluate its safety and toxicity profile. By synthesizing fragmented data, this review provides an evidence-based framework for considering *M. oleifera* as a potential adjunctive therapy for PUD.

## 2 Methods

This systematic review and meta-analysis were carried out according to the Preferred Reporting Items for Systematic Review and Meta-Analyses (PRISMA) Extension Statement (Supplementary File 1) (Page et al., 2021).

### 2.1 Protocol registration

This review was prospectively registered in the International Prospective Register of Systematic Reviews (PROSPERO) on 24 June 2025 (registration number: CRD420251080346). Before submission, we conducted a comprehensive PROSPERO search (23 June 2025) using the keywords *“Moringa oleifera,” “peptic ulcer,” “gastric ulcer,”* and *“drumstick tree.”* No existing or ongoing systematic reviews addressing the therapeutic effects of *M. oleifera* in ulcer treatment were identified. The absence of duplicate registrations highlights the novelty of this work and supports its value in consolidating preclinical evidence on *M. oleifera* for ulcer management.

### 2.2 Eligibility criteria

Eligibility criteria were established according to the PICOS (Population, Intervention, Comparator, Outcomes, and Study Design) framework (see Table 1). The review included preclinical studies conducted on animal models, such as rats, in which ulcers were experimentally induced. To qualify, studies must have evaluated the therapeutic effects of *Moringa oleifera* administered in any form, including extracts, essential oils, or fractions derived from different plant parts such as the leaves, seeds, bark, roots, or flowers. The intervention had to be designed to assess the gastroprotective or ulcer-healing properties of *M. oleifera*.

**TABLE 1:** PICO framework.

PICO element	Description
Population (P)	Humans and animals diagnosed with Peptic Ulcer Disease (PUD), particularly
Intervention (I)	Treatment with *Moringa oleifera* (e.g., leaf, seed, bark, or root extracts/formulations) administered for the purpose of ulcer healing or gastroprotection
Comparator (C)	Standard therapies for PUD, such as proton pump inhibitors (PPIs), histamine-2 receptor antagonists (H2RAs) or placebo treatments
Outcomes (O)	Primary Outcome: - Ulcer healing (e.g., mucosal regeneration, restoration of gastric lining). Secondary Outcomes: - Reduction in ulcer size - Ulcer index reduction
Study design (S)	Randomized controlled trials (RCTs) and preclinical experimental studies (*in vivo* animal models), with quantitative outcomes assessing the efficacy or safety of *Moringa oleifera*

Eligible studies were required to include a comparator group, which could consist either of placebo-treated animals or those receiving established anti-ulcer drugs, such as proton pump inhibitors (omeprazole, esomeprazole, rabeprazole, pantoprazole, or lansoprazole) or histamine-2 receptor antagonists (cimetidine, famotidine, nizatidine, or ranitidine) (Malfertheiner et al., 2009; van Gestel et al., 2024; Wang et al., 2024). The primary outcome of interest was the ulcer index, typically determined by macroscopic scoring of gastric lesions, ulcer area measurement, or other validated severity indices. Secondary outcomes included gastric pH, histopathological findings, oxidative stress markers, mucosal protection, and healing rates as reported in individual studies.

Only randomized controlled trials and preclinical experimental studies published in peer-reviewed journals were considered eligible, provided they reported quantifiable outcomes relevant to ulcer healing or gastroprotection. Studies were excluded if they were observational in nature, case reports, narrative reviews, editorials, letters to the editor, or conference abstracts lacking full data. Research that did not specifically investigate *Moringa oleifera* or failed to report relevant ulcer-related outcomes was also excluded. Finally, qualitative-only studies and those without adequate methodological detail for quality assessment were not considered.

### 2.3 Search strategy

A comprehensive literature search was conducted across five major electronic databases to identify relevant studies evaluating the therapeutic effects of *Moringa oleifera* in the treatment of ulcers. The databases searched included PubMed, Embase (*via* Ovid), Scopus, Web of Science, and the Cochrane Library. The search was supplemented with a manual check of reference lists of included studies and relevant reviews.

The search strategy employed a combination of Medical Subject Headings (MeSH) terms and free-text keywords, which included “*Moringa oleifera*,” “drumstick tree,” “ulcer,” “gastric ulcer,” “peptic ulcer,” “ulcer index,” “gastroprotection,” “gastric mucosal protection,” “anti-ulcerogenic,” and “oxidative stress.” Boolean operators (“AND,” “OR”) were used to combine and refine search terms (Supplementary File 2). Search filters were applied to restrict the results to studies published from 1 January 2000, to 24 June 2025. There were no restrictions on publication status or geographic location, although only English-language studies were included. Grey literature and unpublished studies were not considered in this review.

### 2.4 Study selection and data extraction

The screening process was performed independently and in a blinded manner by two reviewers to minimize selection bias. Disagreements regarding study eligibility were resolved through a third reviewer. A standardized data extraction table was used to collect relevant information from the included studies. Extracted data included the first author’s name, year of publication, animal model used, weight and number of animals, ulcer model employed, type and dose of *Moringa oleifera* extract or formulation, route and duration of administration, comparator/control used, primary and secondary outcomes (e.g., ulcer index, histopathological findings), and any reported adverse effects or toxicity profiles. The data extraction was also carried out by two independent reviewers, and discrepancies were resolved by a third reviewer to ensure accuracy and consistency. This rigorous screening and extraction process laid a strong foundation for the qualitative and quantitative synthesis of the findings.

### 2.5 Risk of bias and data quality assessment

The methodological quality of the included animal studies was evaluated using the SYRCLE (Systematic Review Centre for Laboratory Animal Experimentation) Risk of Bias (RoB) tool, which is an adaptation of the Cochrane RoB tool designed specifically for preclinical research (Hooijmans et al., 2014). This assessment covered nine domains, including the appropriateness of the sample frame, random allocation of animals to groups, adequacy of sample size, detailed description of animals and study settings, coverage of data analysis, validity and reliability of ulcer induction and assessment methods, consistency of outcome measurement across groups, and the appropriateness of statistical analyses (Supplementary File 5). Each study was scored across these domains, with higher scores reflecting a lower risk of bias. Studies that provided detailed methodology, sufficient sample sizes, valid measurement techniques, and robust statistical analysis were classified as having a low risk of bias, thereby strengthening the reliability, reproducibility, and internal validity of their findings.

To evaluate the certainty of evidence, we applied the Grading of Recommendations, Assessment, Development, and Evaluation (GRADE) framework (Pollock et al., 2016). Although traditionally used for clinical studies, GRADE was adapted here for preclinical animal research. The following domains were considered: risk of bias, inconsistency of findings, indirectness of evidence, imprecision of effect estimates, and potential publication bias. Each domain was independently assessed by two reviewers, and the certainty of evidence for each outcome was rated as high, moderate, low, or very low. The final ratings were summarized in a “Synthesis of Findings” table (Table 5).

### 2.6 Statistical analysis

For each included study, we extracted the mean, standard error of the mean (SEM), and sample size for both the control and experimental groups. Standard deviations were calculated using the formula: SD = SEM × $\sqrt{SampleSize}$ .

For ease of analysis, studies were grouped based on the administered doses of *Moringa oleifera* extract: doses ≤350 mg/kg body weight were classified as low, while doses ≥400 mg/kg body weight were classified as high (Supplementary File 4). Effect sizes were standardized as standard mean difference (SMD), employing Hedge’s g to adjust for potential bias associated with small sample sizes due to the limited number of studies.

Given the observed variability among the studies, we applied a random-effects meta-analysis model using the DerSimonian-Laird estimator. We assessed statistical heterogeneity with the I^2^ statistic, which quantifies the degree of variation across studies. Values of approximately 25%, 50%, and 75% were interpreted as low, moderate, and high heterogeneity, respectively.

To evaluate the potential for publication bias, we conducted Egger’s regression test and performed a trim-and-fill analysis. Subgroup analyses were limited to factors represented by at least three studies to ensure adequate statistical power. Sensitivity analyses were carried out to investigate the influence of outlier studies on the overall pooled effect. If the exclusion of any outlier significantly altered the pooled SMD, those studies were removed prior to conducting subgroup analyses.

Results were visually summarized using forest plots to present the overall effect sizes, alongside funnel plots that illustrated the outcomes of the trim-and-fill analyses. Statistical significance was defined as a p-value of less than 0.05. All statistical analyses were conducted in R (version 4.4.2) utilising the metafor and meta packages.

## 3 Results

### 3.1 Search result

**FIGURE 1:**
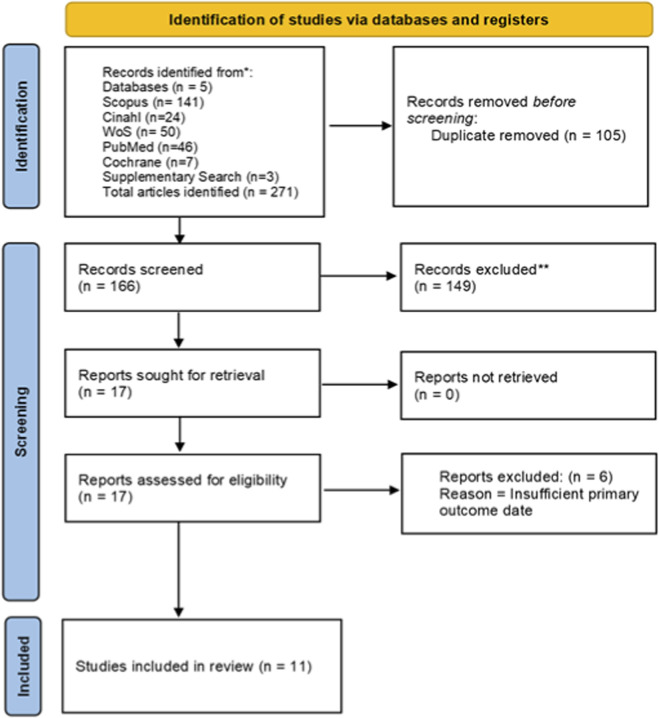
PRISMA flowchart for study selection.

A total of 271 records were retrieved from the following databases: Scopus (141), CINAHL (24), Web of Science (50), PubMed (46), Cochrane Library (7), and freehand searches (3). After removing 105 duplicates, 166 unique records remained for screening (Figure 1). The study selection process followed PRISMA 2020 guidelines, with the flow of studies detailed in the PRISMA diagram (Figure 1) (Page et al., 2021).

Screening of titles and abstracts led to the exclusion of 149 studies that did not meet the eligibility criteria. Full-text review was conducted for the remaining 17 articles. Of these, three were excluded because their reported data did not align with the review outcomes. An additional freehand search yielded five studies, of which three were eligible and added, bringing the total to 17 studies considered at this stage (Supplementary File 3).

During data extraction, six studies (Akhtar et al., 2021; Bapan et al., 2022; Debnath et al., 2011; Mabrok and Mohamed, 2019; Olajide et al., 2024; Ruckmani et al., 1998) were excluded due to insufficient primary outcome data. This left 11 studies (Devaraj et al., 2007; Alissa et al., 2025; Choudhary et al., 2013; Lawal et al., 2018; Debnath et al., 2011; Abdu and Garba, 2021; Airaodion et al., 2019; Dahiru et al., 2006; Das et al., 2011; Debnath and Guha, 2007; Patel et al., 2018) encompassing 268 animals, which were included in the final qualitative and quantitative synthesis (Supplementary Files 3 and 4). No risks of bias were identified from missing data in the included studies.

### 3.2 Characteristics of included studies

The 11 included studies were conducted primarily in India (Devaraj et al., 2007; Choudhary et al., 2013; Debnath et al., 2011; Das et al., 2011; Debnath and Guha, 2007; Patel et al., 2018) and Nigeria (Alissa et al., 2025; Lawal et al., 2018; Abdu and Garba, 2021; Airaodion et al., 2019; Dahiru et al., 2006) and consistently demonstrated the anti-ulcer potential of *Moringa oleifera* across diverse experimental models, including pylorus ligation (Alissa et al., 2025; Patel et al., 2018), ethanol (Choudhary et al., 2013), indomethacin (Lawal et al., 2018; Abdu and Garba, 2021; Airaodion et al., 2019; Dahiru et al., 2006), aspirin (Debnath et al., 2011), ibuprofen (Das et al., 2011), and stress-induced ulcers (Debnath and Guha, 2007). The animal models used were mainly Wistar (Devaraj et al., 2007; Alissa et al., 2025; Choudhary et al., 2013; Lawal et al., 2018; Debnath et al., 2011; Abdu and Garba, 2021; Airaodion et al., 2019; Dahiru et al., 2006; Das et al., 2011; Debnath and Guha, 2007; Patel et al., 2018).

Different plant parts, leaves (Devaraj et al., 2007; Alissa et al., 2025; Debnath et al., 2011; Abdu and Garba, 2021; Airaodion et al., 2019; Dahiru et al., 2006; Das et al., 2011; Debnath and Guha, 2007), seeds (Lawal et al., 2018), root bark (Choudhary et al., 2013), flowers (Patel et al., 2018), and fruits (Devaraj et al., 2007), were tested in various extract forms such as aqueous (Lawal et al., 2018; Debnath et al., 2011; Airaodion et al., 2019; Dahiru et al., 2006; Das et al., 2011; Debnath and Guha, 2007), methanol (Abdu and Garba, 2021), ethanol (Alissa et al., 2025; Choudhary et al., 2013), acetone (Devaraj et al., 2007; Patel et al., 2018), chloroform (Devaraj et al., 2007), and petroleum ether (Devaraj et al., 2007; Patel et al., 2018), administered at varying doses. Across studies, *M. oleifera* significantly reduced ulcer index (Devaraj et al., 2007; Alissa et al., 2025; Choudhary et al., 2013; Lawal et al., 2018; Debnath et al., 2011; Abdu and Garba, 2021; Airaodion et al., 2019; Dahiru et al., 2006; Das et al., 2011; Debnath and Guha, 2007; Patel et al., 2018), gastric volume (Lawal et al., 2018), and acidity (Devaraj et al., 2007; Alissa et al., 2025; Choudhary et al., 2013; Lawal et al., 2018; Abdu and Garba, 2021; Das et al., 2011; Patel and Lariya, 2019), while increasing gastric pH (Devaraj et al., 2007; Alissa et al., 2025; Choudhary et al., 2013; Lawal et al., 2018; Abdu and Garba, 2021; Das et al., 2011), mucosal thickness (Devaraj et al., 2007; Choudhary et al., 2013; Dahiru et al., 2006; Patel and Lariya, 2019), mucin secretion (Devaraj et al., 2007; Patel and Lariya, 2019), and antioxidant enzyme activity (Devaraj et al., 2007) (e.g., SOD, CAT). Histological assessments also confirmed mucosal protection (Devaraj et al., 2007; Das et al., 2011; Debnath and Guha, 2007) and healing (Abdu and Garba, 2021).

In several cases, the effects of *M. oleifera* were comparable to standard anti-ulcer drugs such as cimetidine (Alissa et al., 2025; Lawal et al., 2018; Abdu and Garba, 2021), omeprazole (Choudhary et al., 2013; Lawal et al., 2018; Airaodion et al., 2019), ranitidine (Debnath and Guha, 2007; Patel and Lariya, 2019), and famotidine (Das et al., 2011), though sometimes slightly less pronounced. The gastroprotective effects were dose-dependent (Lawal et al., 2018; Abdu and Garba, 2021; Dahiru et al., 2006), with some extracts achieving up to 95% ulcer inhibition alongside improvements in mucosal integrity (Alissa et al., 2025; Choudhary et al., 2013; Dahiru et al., 2006) and serotonergic cell recovery (Debnath et al., 2011).

Overall, these findings strongly support the gastroprotective and anti-ulcerogenic potential of *M. oleifera*. The detailed characteristics of all included studies are summarized in Table 2.

**TABLE 2:** Characteristics of included studies.

S/N	Author (Year)	Country	Study design	Ulcer model(s)	Animal model (weight, n)	Moringa intervention	Control/Standard drug	Key outcomes	References
1	Devaraj et al. (2007)	India	RCT	Acetic acid, pylorus ligation, indomethacin, ethanol, cold-restraint stress, cysteamine	Wistar rats (200–250 g, n = 5–6)	Leaves and fruit; methanol, acetone, chloroform, petroleum ether extracts (500 mg/kg)	Normal saline/No standard drug stated	↓Ulcer index, ↑pH, ↓acidity, ↑SOD, CAT, mucosal thickness, mucin, gland width, histological protection	Devaraj et al. (2007)
2	Dahiru et al. (2006)	Nigeria	RCT	Indomethacin	Albino rats (160–170 g, n = 20)	Leaf aqueous extract (100–400 mg/kg)	100 mg/kg indomethacin (no standard drug)	Dose-dependent ↓ulcer index (up to 95.3% inhibition), mucosal protection	Dahiru et al. (2006)
3	Alissa et al. (2025)	Nigeria	RCT	Pylorus ligation	Wistar rats (150–200 g, n = 40)	Leaf ethanol fraction (K3, 200 mg/kg)	Normal saline/Cimetidine (32 mg/kg)	Ulcer index ↓ to 0.24, ↑pH (4.09), ↓total acidity, 92.75% protection	Alissa et al. (2025)
4	Lawal et al. (2018)	Nigeria	RCT	Pylorus ligation, Indomethacin	Wistar rats (120–150 g, n = 30)	Seed aqueous extract (500, 1,000, 1,500 mg/kg)	Distilled water/Omeprazole, Cimetidine	↓Ulcer index, ↑pH, ↓gastric volume, ↓acidity, dose-dependent effect	Lawal et al. (2018)
5	Das et al. (2011)	India	RCT	Pylorus ligation, Ibuprofen	Wistar rats (180–200 g, n = 48)	Leaf aqueous extract (200, 400 mg/kg)	1% CMC/Famotidine (3.6 mg/kg)	↓Ulcer index, ↓free/total acidity, ↑pH, histological protection	Das et al. (2011)
6	Choudhary et al. (2013)	India	RCT	Ethanol, Pylorus ligation	Wistar rats (200–250 g, n = 30)	Root bark methanol extract (150–500 mg/kg)	Saline/Omeprazole (30 mg/kg)	Up to 86.15% ulcer inhibition, ↓acidity, ↑pH, improved mucosal integrity	Choudhary et al. (2013)
7	Debnath and Guha (2007)	India	RCT	Aspirin, Cold stress, Cerebellar lesion	Holtzman rats (150–200 g, n = 72)	Leaf aqueous extract (300 mg/kg)	Saline	↑EC cell count, ↑5-HT, ↓ulcer index, histological recovery	Debnath and Guha (2007)
8	Debnath et al. (2011)	India	RCT	Aspirin	Holtzman rats (150–200 g, n = 48)	Leaf aqueous extract (300 mg/kg)	Saline/Ranitidine	↓Ulcer index, ↑5-HT, ↑EC cell density, effective gastroprotection	Debnath et al. (2011)
9	Patel et al. (2018)	India	RCT	Pylorus ligation	Wistar rats (200–250 g, n = NA)	Flower extract (petroleum ether, acetone, methanol at 500 mg/kg)	Ranitidine (50 mg/kg)	↓Free/total acidity, ↑mucin, ↑mucosal thickness, modest ulcer index reduction	Patel et al. (2018)
10	Abdu and Garba (2021)	Nigeria	RCT	Indomethacin	Swiss albino mice (120–150 g, n = 30)	Leaf methanol extract (200–800 mg/kg)	Distilled water/Cimetidine	Dose-dependent ↓ulcer index, ↓acidity, ↑healing, ↑pH	Abdu and Garba (2021)
11	Airaodion et al. (2019)	Nigeria	RCT	Indomethacin (7/14 days)	Albino rats (150–200 g, n = 30)	Leaf aqueous solution (0.8 mg/mL)	Distilled water/Omeprazole	↓Ulcer score by 53.43%–57.58%, < omeprazole, effective preventive effect	Airaodion et al. (2019)

n, number of subjects per experimental group; ↓, decrease; ↑increase; <, greater than; RCT, randomised control trial; CMC, carboxy methyl cellulose; SOD, superoxide dismutase.

### 3.3 Meta-analysis

Studies were stratified into high-dose (Supplementary File 4) and low-dose (Supplementary File 4) groups based on the administered dosage of *Moringa oleifera* extracts. Separate meta-analyses were conducted for comparisons with basal controls and standard drug controls.

#### 3.3.1 High-dose group

**FIGURE 2:**
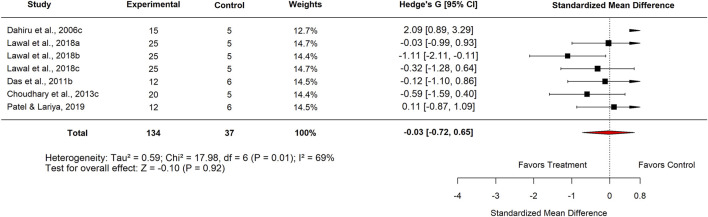
Effect sizes for included studies comparing high dose moringa extract with standard drug.

Seven studies compared *M. oleifera* extracts at high doses with standard anti-ulcer drugs. The pooled standard mean difference (SMD) was −0.03 (95% CI: −0.27 to 0.66; p = 0.92) (Figure 2; Supplementary File 7), indicating no significant difference overall. However, heterogeneity across studies was considerable (I^2^ = 69%). While *M. oleifera* tended to reduce ulcer index slightly compared to standard drugs, the effect was not statistically significant.

**FIGURE 3:**
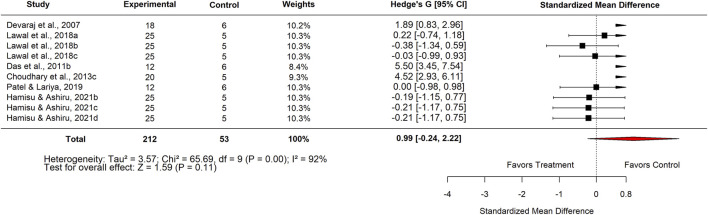
Standard mean difference (Hedges’ g) with 95% confidence intervals comparing extract of Moringa to the basal control.

**FIGURE 4:**
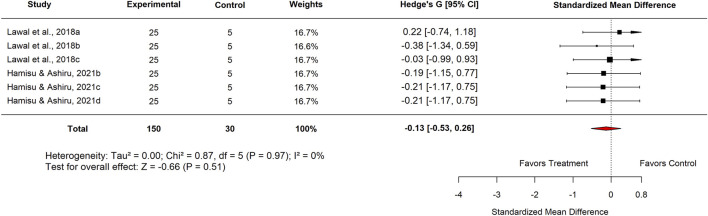
Standard mean difference (Hedges’ g) with 95% confidence intervals comparing high doses of Moringa leaf extract to basal control.

**FIGURE 5:**
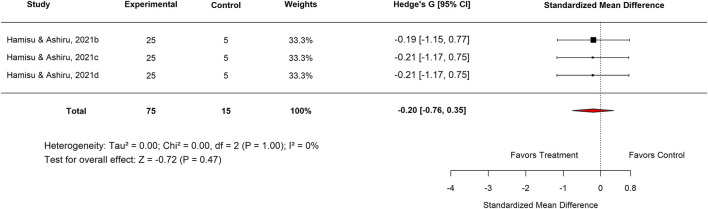
Standard mean difference (Hedges’ g) with 95% confidence intervals comparing methanol Moringa extract to basal control.

**FIGURE 6:**
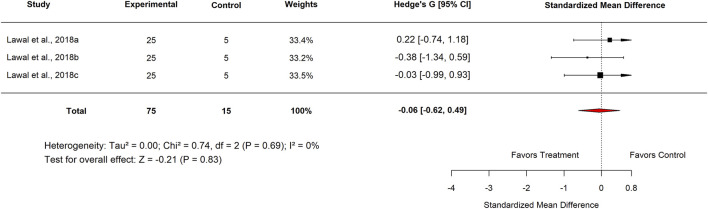
Standard mean difference (Hedges’ g) with 95% confidence intervals comparing aqueous seed extract of Moringa to standard drug.

Ten studies compared *M. oleifera* with basal controls. The pooled SMD was 0.99 (95% CI: 0.24–2.22; p = 0.11) (Figures 3–6), also showing no significant difference.

**FIGURE 7:**
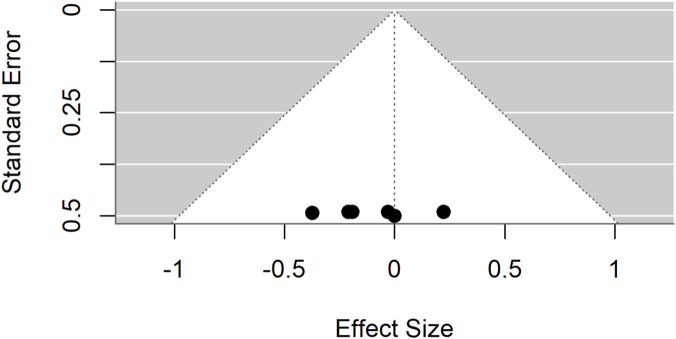
Standard mean difference (Hedges’ g) with 95% confidence interval comparing *Moringa oleifera* to basal control.

**FIGURE 8:**
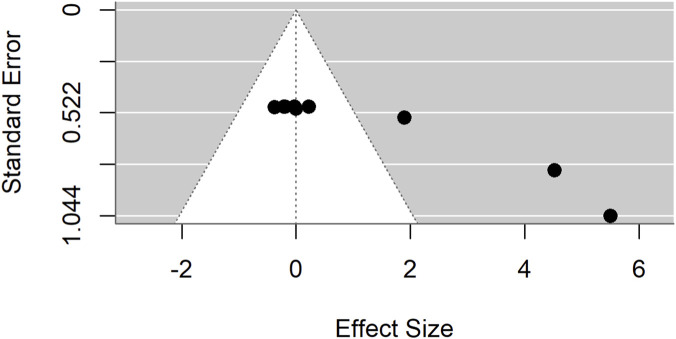
Egger’s test and trim-and-fill analyses revealing publication bias for standard drug comparison.

**FIGURE 9:**
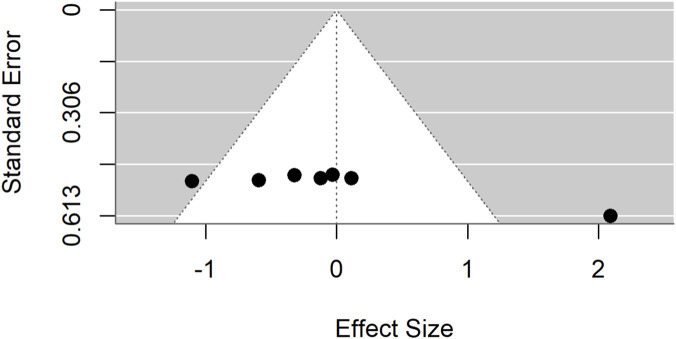
Egger’s test and trim-and-fill analyses reveal publication bias for basal control comparison.

**FIGURE 10:**
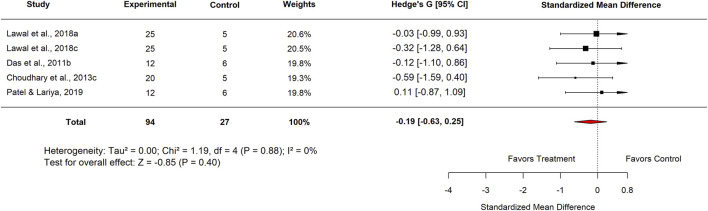
Standard mean difference (Hedges’ g) with 95% confidence interval comparing doses of *Moringa oleifera* to standard drug.

**FIGURE 11:**
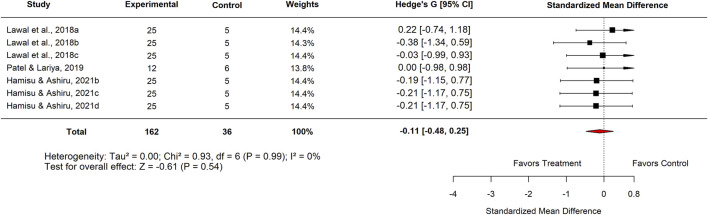
Standard mean difference (Hedges’ g) with 95% confidence interval comparing doses of *Moringa oleifera* to basal control.

Publication bias was evident in both comparisons: Egger’s test p = 0.0017 for standard drugs and p < 0.0001 for basal controls. Sensitivity analyses excluding an outlier (Figures 7–9) resolved this bias. After exclusion, the adjusted pooled SMD for standard drugs was −0.19 (95% CI: −0.63 to 0.25; p = 0.40), with no heterogeneity (I^2^ = 0%) or bias (Egger’s p = 0.66). For basal controls, the adjusted SMD was −0.11 (95% CI: −0.48 to 0.25; p = 0.54), again with no heterogeneity (I^2^ = 0%) or bias (Egger’s p = 0.89) (Figures 10, 11).

##### 3.3.1.1 Subgroup analysis for high-dose strata

**FIGURE 12:**
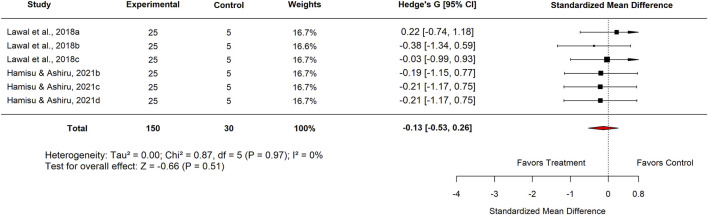
Standard mean difference (Hedges’ g) with 95% confidence intervals comparing Moringa extract to standard drug.

**FIGURE 13:**
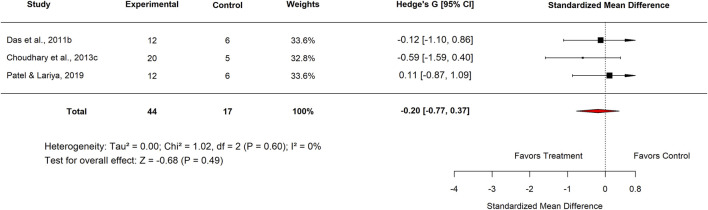
Standard mean difference (Hedges’ g) with 95% confidence intervals comparing aqueous extract of Moringa to the standard drug.

**FIGURE 14:**
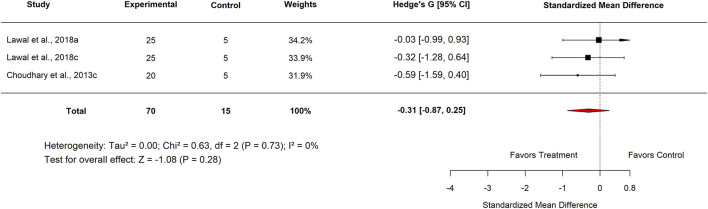
Standard mean difference (Hedges’ g) with 95% confidence intervals comparing Moringa extract to basal control.

The subgroup analysis evaluated the effects of Moringa across various categories. In the Standard Drug subgroup, Nigeria studies reported an SMD of −0.20 (95% CI: −0.77 to 0.37; p = 0.49) (Figure 12), showing no significant difference between *M. oleifera* and standard drugs. For the aqueous extract subgroup, the pooled SMD was −0.31 (95% CI: −0.87 to 0.25; p = 0.28) (Figures 13, 14), also indicating no statistically significant effect.

Within the Basal Control subgroup, Nigerian studies reported an SMD of −0.46 (95% CI: −0.91 to −0.01; p = 0.51), which did not reach statistical significance. Similarly, analysis by plant part showed that leaf extracts produced an SMD of −0.13 (95% CI: −0.53 to 0.26; p = 0.51) (Figure 14), also indicating no meaningful difference compared with basal controls.

**FIGURE 15:**
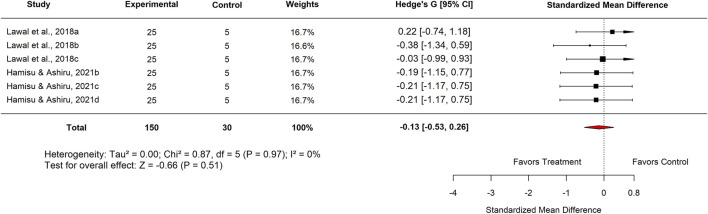
Standard mean difference (Hedges’ g) with 95% confidence intervals comparing Moringa extract to Indomethacin.

**FIGURE 16:**
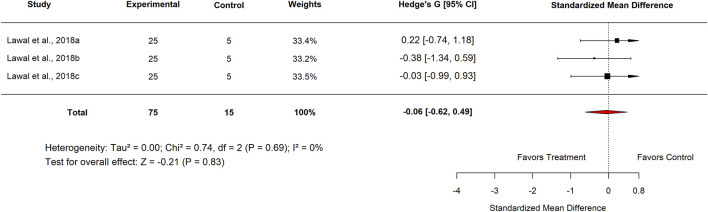
Standard mean difference (Hedges’ g) with 95% confidence intervals comparing Moringa seed extract to basal control.

In the Experimental Model subgroup, *Moringa oleifera* extracts tested against indomethacin-induced ulcers showed no significant effect (SMD = −0.13; 95% CI: −0.53 to 0.26; p = 0.83) (Figure 15). Similarly, aqueous extracts produced a non-significant effect (SMD = −0.06; 95% CI: −0.62 to 0.49; p = 0.84) (Figure 16). By contrast, methanol extracts demonstrated a significant protective effect, with an SMD of −0.62 (95% CI: −1.06 to −0.17; p = 0.01), favouring *M. oleifera* (Table 2).

Table 3 summarizes the subgroup analysis of the anti-ulcer effects of high doses of *moringa oleifera* extracts compared with controls.

**TABLE 3:** Subgroup analysis of the anti-ulcer effects of high doses of *Moringa oleifera* extracts compared with controls.

Control	Subgroup	Levels	No. of studies	Hedge’s G (95% CI)	I^2^ (%)	P-value
Standard drug	Country	Nigeria	3	−0.20 (−0.77–0.37)	0	0.49
	Extract tested	Aqueous	3	−0.16 (−0.72–0.40)	0	0.58
	Standard drug	Proton pump inhibitor Omeprazole 20 mg/kg	3	−0.31 (−0.87–0.25)	0	0.28
Basal	Country	Nigeria	6	−0.13 (−0.53–0.26)	0	0.51
	Plant part used	Leaf	6	−0.13 (−0.53–0.26)	0	0.51
		Plant	3	−0.06 (−0.62–0.49)	0	0.83
	Experimental model used	Indomethacin-induced gastric ulcer	6	−0.13 (−0.53–0.26)	0	0.51
	Extract tested	Aqueous	3	−0.06 (−0.62–0.49)	0	0.83
		Methanol	3	−0.20 (−0.76–0.35)	0	0.47

#### 3.3.2 Low-dose group

**FIGURE 17:**
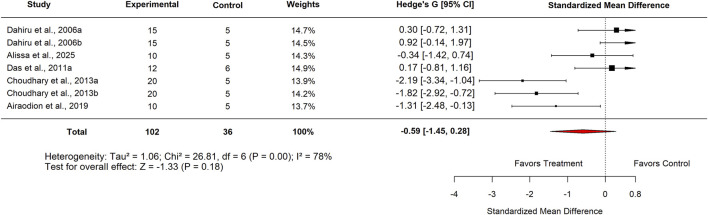
Standard mean difference (Hedges’ g) with 95% confidence intervals comparing low doses Moringa extract to standard drug control.

A meta-analysis was also performed to evaluate the efficacy of *Moringa oleifera* at low doses. When compared with standard drug controls, the pooled SMD was −0.59 (95% CI: –1.45 to 0.28; p = 0.18) (Figure 17), showing no statistically significant difference. Considerable heterogeneity was observed across studies (I^2^ = 78%). Evidence of publication bias was detected through both Egger’s test (p < 0.001) and trim-and-fill analyses.

**FIGURE 18:**
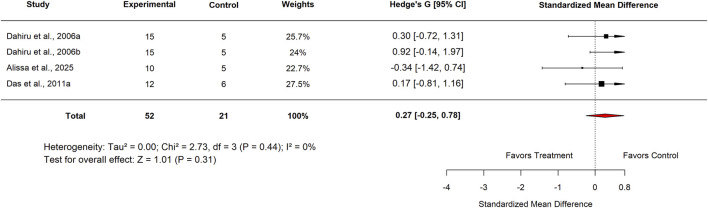
Standard mean difference (Hedges’ g) with 95% confidence interval comparing sensitivity effect between Moringa extract to standard drug.

Following the removal of outlier studies (references to be specified), heterogeneity was eliminated (I^2^ = 0%). However, the effect remained non-significant, with an adjusted pooled SMD of 0.27 (95% CI: −0.25 to 0.78; p = 0.31) (Figure 18).

**FIGURE 19:**
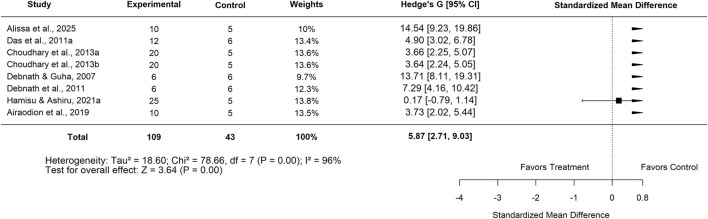
Standard mean difference (Hedges’ g) with 95% confidence intervals comparing low doses of Moringa extract to basal control.

**FIGURE 20:**
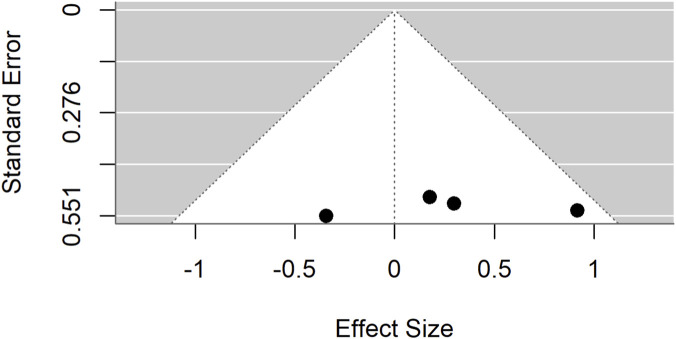
Sensitivity trim-and-fill analyses revealing publication bias for low dose *Moringa oleifera* compared to standard drug.

**FIGURE 21:**
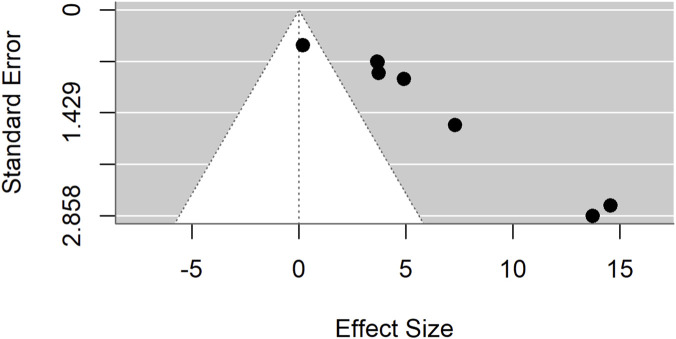
Egger’s test and trim-and-fill analyses revealing publication bias for low dose *Moringa oleifera* compared to basal control.

**FIGURE 22:**
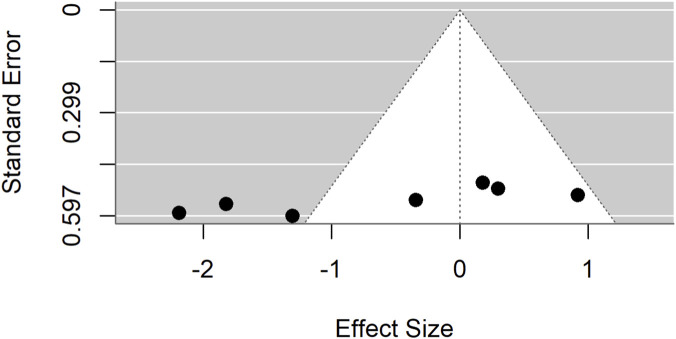
Egger’s test and trim-and-fill analyses revealing publication bias for low dose *Moringa oleifera* compared to standard drug.

In contrast, comparisons between *Moringa oleifera* and basal controls yielded a pooled SMD of 5.87 (95% CI: 2.71–9.03; p < 0.001) (Figure 19), demonstrating a significantly greater effect in favor of *Moringa*. This analysis, however, showed very high heterogeneity (I^2^ = 96%). Evidence of publication bias was confirmed by Egger’s test (p = 0.0044) and trim-and-fill analyses (Figures 20–22).

**FIGURE 23:**
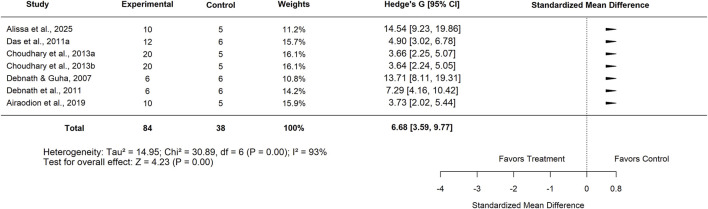
Standard mean difference (Hedges’ g) with 95% confidence intervals comparing low doses of Moringa extract to basal control.

Sensitivity analysis, performed after excluding the identified outlier study, produced a similar pooled effect (SMD = 6.68; 95% CI: 3.59–9.77; p < 0.001) (Figure 23), with heterogeneity slightly reduced but still substantial (I^2^ = 93%). Since removing outliers did not materially change the effect size, further subgroup analyses were conducted using the full dataset.

##### 3.3.2.1 Subgroup analysis

**FIGURE 24:**
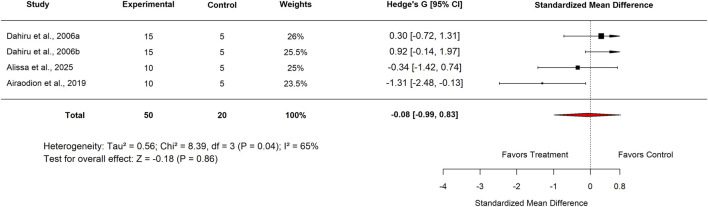
Effect sizes for included studies comparing low doses of Moringa extract with standard drug in Nigeria.

**FIGURE 25:**
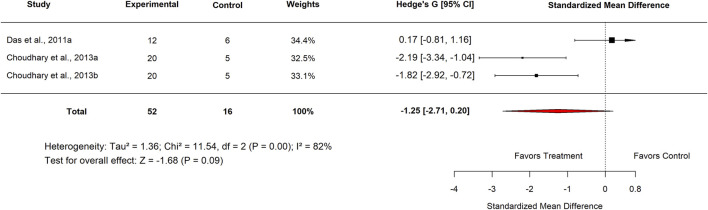
Effect sizes for included studies comparing low doses of Moringa extract with standard drug in India.

In the standard drug comparison, subgroup analysis by country showed no significant differences in efficacy between *Moringa oleifera* and standard drugs. Studies conducted in Nigeria reported an SMD of −0.08 (95% CI: −0.99 to 0.83; p = 0.86) with moderate heterogeneity (I^2^ = 65%) (Figure 24). Similarly, studies from India yielded an SMD of −1.25 (95% CI: −2.71 to 0.20; p = 0.09) with high heterogeneity (I^2^ = 82%) (Figure 25). These findings indicate that, across both countries, *M. oleifera* did not demonstrate a statistically significant advantage over standard drug therapies.

##### 3.3.2.2 Plant part and extract type analyses

**FIGURE 26:**
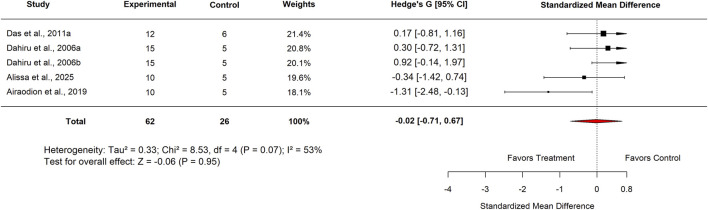
Standard mean difference (Hedges’ g) with 95% confidence intervals comparing low doses of Moringa leaf extract to standard drug.

**FIGURE 27:**
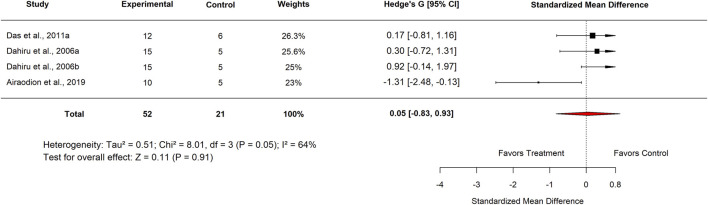
Standard mean difference (Hedges’ g) with 95% confidence intervals comparing low doses of aqueous Moringa extract to standard drug.

When analyzed by plant part, studies using *Moringa oleifera* leaf extract reported an SMD of −0.02 (95% CI: −0.71 to 0.67; p = 0.95) with moderate heterogeneity (I^2^ = 53%) (Figure 26), indicating no significant effect. Similarly, analysis by extract type showed that aqueous extracts had an SMD of 0.05 (95% CI: −0.83 to 0.93; p = 0.91) with substantial heterogeneity (I^2^ = 64%) (Figure 27), also revealing no significant difference in efficacy.

##### 3.3.2.3 Comparison with omeprazole

**FIGURE 28:**
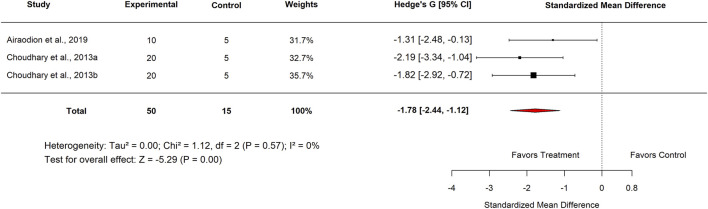
Standard mean difference (Hedges’ g) with 95% confidence intervals of low dose standard drug.

**FIGURE 29:**
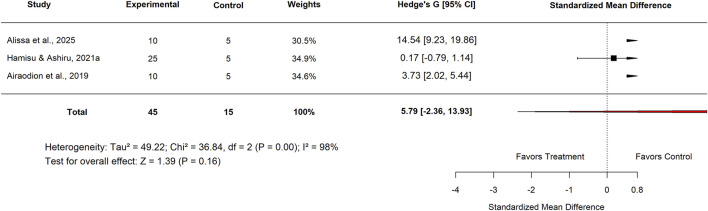
Standard mean difference (Hedges’ g) with 95% confidence intervals for low basal control doses in Nigeria.

In the subgroup analysis comparing *Moringa oleifera* with the proton pump inhibitor omeprazole, three studies reported a pooled SMD of −1.78 (95% CI: −2.44 to −1.12; p < 0.001) (Figures 28, 29). No heterogeneity was observed (I^2^ = 0%). These results indicate that *M. oleifera* is significantly less effective than omeprazole in reducing ulcer severity.

##### 3.3.2.4 Basal control comparisons by country

**FIGURE 30:**
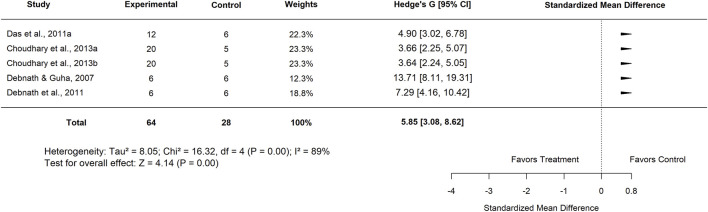
Standard mean difference (Hedges’ g) with 95% confidence intervals for low basal control doses in India.

In the basal control subgroup, studies from Nigeria reported an SMD of 5.79 (95% CI: −2.36 to 13.93; p = 0.16) with extremely high heterogeneity (I^2^ = 98%), indicating no statistically significant effect. In contrast, studies conducted in India showed a strong positive effect, with an SMD of 5.85 (95% CI: 3.08–8.62; p < 0.001) and substantial heterogeneity (I^2^ = 89%) (Figure 30). These findings suggest that while results from Nigeria were inconclusive, Indian studies consistently demonstrated significant efficacy of *Moringa oleifera* compared with basal controls.

##### 3.3.2.5 Stratification by plant part and extract type

**FIGURE 31:**
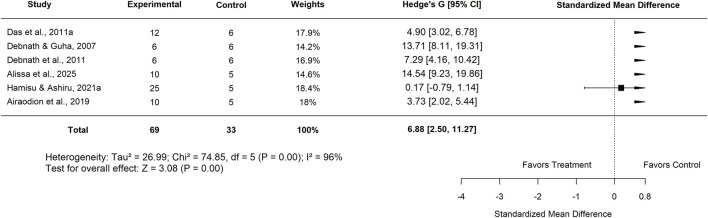
Standard mean difference (Hedges’ g) with 95% confidence intervals for comparison of low dose Moringa leaf and basal control.

**FIGURE 32:**
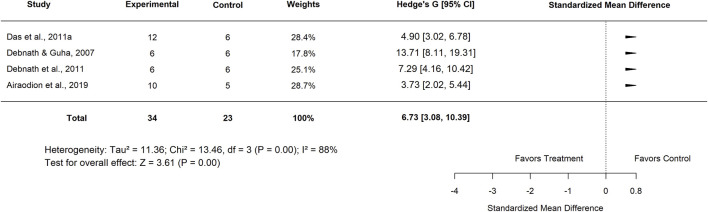
Standard mean difference (Hedges’ g) with 95% confidence intervals for comparison of low dose aqueous extract of Moringa leaf and basal control.

Further subgroup analysis by plant part showed that studies using the leaf extract reported a pooled SMD of 6.88 (95% CI: 2.50–11.27; p < 0.001) (Figure 31), with very high heterogeneity (I^2^ = 96%), indicating a significant beneficial effect. Similarly, studies evaluating aqueous extracts yielded an SMD of 6.73 (95% CI: 3.08–10.39; p < 0.001) (Figure 32), with high heterogeneity (I^2^ = 88%), further supporting the efficacy of *Moringa oleifera* in this form.

Table 4 presents the subgroup analysis of low-dose *M. oleifera* extracts compared with standard drugs and basal controls in experimental ulcer models, while Table 5 provides a synthesis of findings across all included studies.

**TABLE 4:** Subgroup analysis of low doses of *Moringa oleifera* extracts versus standard drugs and basal controls in experimental ulcer models.

Control	Subgroup	Levels	No. of studies	Hedge’s G (95% CI)	I^2^ (%)	P-value
Standard drug	Country	Nigeria	4	−0.08 (−0.99–0.83)	65	0.86
		India	3	−1.25 (−2.71–0.20)	82	0.09
	Plant	Leaf	5	−0.02 (−0.71–0.67)	53	0.95
	Extract tested	Aqueous	4	0.05 (−0.83–0.93)	64	0.91
	Standard drug	Proton pump inhibitor omeprazole	3	−1.78 (−2.44–1.12)	0	<0.001
Basal	Country	Nigeria	3	5.79 (−2.36–13.93)	98	0.16
		India	5	5.85 (3.08–8.62)	89	<0.001
	Plant part used	Leaf	6	6.88 (2.50–11.27)	96	<0.001
	Extract tested	Aqueous	4	6.73 (3.08–10.39)	88	<0.001

**TABLE 5:** Synthesis of findings from included studies.

Author (year)	Primary outcomes (ulcer healing, ulcer index)	Secondary outcomes (biochemical markers, histology, side effects)	References
Devaraj et al. (2007)	↓Ulcer index across all models; methanol group: 4.0 ± 1.05; up to 85% protection	↑pH (6.92–7.66), ↓gastric volume/acidity, ↑SOD, catalase, mucin, mucosal thickness, ↑glandular width; histological healing confirmed; no toxicity reported	Devaraj et al. (2007)
Dahiru et al. (2006)	Dose-dependent ↓ulcer index from 14.6 to 1.0; up to 95.3% protection	Gross mucosal preservation; no side effects noted; no biochemical markers reported	Dahiru et al. (2006)
Alissa et al. (2025)	Ulcer index ↓ to 0.24; 92.75% ulcer protection (comparable to cimetidine)	↑pH (4.09), ↓total acidity (28.9 meq/L), histological mucosal healing, anti-*H. pylori* activity; no adverse effects reported	Alissa et al. (2025)
Lawal et al. (2018)	↓Ulcer index (e.g., 3.42 at 1,500 mg/kg); protection: 25%–45% (dose-dependent)	↑pH, ↓gastric volume and acidity; histology: mucosal regeneration; safe at all doses	Lawal et al. (2018)
Das et al. (2023)	↓Ulcer index: 0.0841 at 200 mg/kg (comparable to famotidine)	↑pH, ↓free/total acidity, improved histological integrity; no side effects observed	Das et al. (2023)
Choudhary et al. (2013)	Ulcer index ↓ to 0.68 (500 mg/kg); up to 86.15% protection	↑pH (2.21), ↓acidity, restored mucosal layers histologically; no adverse effects	Choudhary et al. (2013)
Debnath et al. (2011)	↓Ulcer index (e.g., 9.50 ± 0.76); across multiple stress models	↑EC cell count (2633), ↑5-HT (1.32 µg/100 g), enhanced serotonergic healing; no side effects	Debnath et al. (2011)
Debnath et al. (2011)	↓Ulcer index: 10.30 (vs. 38.58 in aspirin-only), close to ranitidine	↑5-HT (84.8), ↑EC cell density, histological protection; safe use demonstrated	Debnath et al. (2011)
Patel et al. (2018)	Modest ulcer index ↓; acetone and methanol extracts showed greater effect than petroleum ether	↓Free/total acidity, ↑mucin content, ↑mucosal thickness; mild effect on ulcer healing; no toxicity reported	Patel et al. (2018)
Abdu and Garba (2021)	Dose-dependent ↓ulcer index (e.g., 3.04 at 800 mg/kg); up to 84% inhibition	↑pH, ↓acidity (24.88 meq/L), histological evidence of mucosal healing; no adverse effects	Abdu and Garba (2021)
Devaraj et al. (2007)	↓Ulcer score: 53.43% (day 7) and 57.58% (day 14); < omeprazole (72.6%–74.29%)	Effective ulcer prevention; no biochemical data; safe on repeated dosing; no toxic effects observed	Airaodion et al. (2019)

## 4 Discussion

Peptic ulcer disease (PUD) continues to be a significant health challenge, particularly in low- and middle-income countries where access to conventional drugs may be limited. In such contexts, medicinal plants have gained increasing attention as affordable and culturally acceptable alternatives or adjunctive therapies (Devaraj et al., 2007; Abdu and Garba, 2021; Chaudhary et al., 2023). Among these, *Moringa oleifera* has been widely studied for its gastroprotective potential (Airaodion et al., 2019; Das et al., 2011; Patel and Lariya, 2019). This systematic review and meta-analysis synthesized eleven preclinical studies (Figure 1; Supplementary File 3) and demonstrated that *M. oleifera* extracts consistently reduced ulcer index, preserved mucosal integrity, and modulated oxidative and inflammatory pathways when compared with untreated controls.

### 4.1 Therapeutic effects and histological findings

Our findings confirm that *M. oleifera* possesses broad gastroprotective and ulcer-healing effects across diverse preclinical models. Histopathological evaluations frequently reported improved epithelial regeneration, reduced necrosis, and restoration of glandular structures in treated animals (Table 2). For example, one study documented significant reductions in ulcer index across six experimental models, with concurrent increases in antioxidant enzyme levels and mucin secretion (Devaraj et al., 2007). Another reported dose-dependent effect, with aqueous extracts achieving more than 95% mucosal protection against indomethacin-induced ulcers (Dahiru et al., 2006). These results validate the ethnopharmacological use of *M. oleifera* in gastrointestinal disorders and suggest its relevance as a potential adjunctive therapy for PUD. Variability in efficacy, however, highlights the role of extraction methods, plant part used, and ulcer model in shaping outcomes.

### 4.2 Bioactive compounds and mechanisms of action

The therapeutic actions of *M. oleifera* appear to be driven by multiple phytochemicals acting synergistically. Leaves contain chlorogenic acid, quercetin, kaempferol, and isothiocyanates, all of which are known to exert gastroprotective effects (Abdu and Garba, 2021; Airaodion et al., 2019; Dahiru et al., 2006). These compounds modulate inflammatory mediators, inhibit lipid peroxidation, scavenge free radicals, and restore mucosal integrity (Jo et al., 2020; Serafim et al., 2020). Isothiocyanates, for instance, enhance nitric oxide synthase activity, promoting vasodilation, angiogenesis, and tissue repair (Fahey, 2017). Flavonoids such as quercetin stabilize mast cells and suppress histamine release, thereby reducing gastric acid hypersecretion and motility (Pareek et al., 2023). Experimental studies support these mechanisms: one ethanol fraction (K3) inhibited gastric acid secretion and provided >90% ulcer protection, with histological evidence of epithelial restoration (Alissa et al., 2025). Collectively, these findings emphasize that the gastroprotective effect of *M. oleifera* cannot be attributed to a single compound but rather to synergistic interactions across multiple pathways.

### 4.3 Influence of extract type and plant part

Differences in solvent extraction strongly influenced efficacy. Methanolic extracts tended to produce greater ulcer inhibition than aqueous preparations, likely due to higher flavonoid and phenolic yields (Choudhary et al., 2013; Abdu and Garba, 2021). For example, methanol root bark extract achieved up to 86% ulcer inhibition (Choudhary et al., 2013), compared to lower protection rates for aqueous leaf extracts. Conversely, aqueous seed extracts demonstrated only modest effects (Lawal et al., 2018). These differences suggest that extraction methods and plant part selection are critical to pharmacological potency. Although leaf extracts were most frequently studied and consistently beneficial, comparative analyses across roots, seeds, and flowers remain limited. Standardized extraction protocols and systematic evaluations of plant parts are urgently needed to improve reproducibility.

### 4.4 Comparison with control groups and standard drugs

Our meta-analysis showed that standard drugs such as omeprazole and ranitidine generally outperformed *M. oleifera*, particularly at lower doses. For example, omeprazole conferred 72%–74% protection compared with 57% for aqueous *M. oleifera* extract in a 14-day indomethacin model (Airaodion et al., 2019), while ranitidine also reduced ulcer indices more effectively in some experiments (Debnath et al., 2011). However, ethanol fractions of *M. oleifera* provided protection comparable to cimetidine in certain models (Alissa et al., 2025). These findings indicate that while *M. oleifera* demonstrates robust anti-ulcer activity, it may not universally match the potency of conventional drugs. It is therefore better positioned as an adjunct therapy rather than a direct alternative. Future studies should explore potential synergistic effects of combining *M. oleifera* with standard drugs to optimize efficacy and reduce toxicity.

### 4.5 Safety considerations

Most included studies reported no adverse effects of *M. oleifera*, even at high doses. Extracts administered up to 1,500 mg/kg and 800 mg/kg (Lawal et al., 2018; Abdu and Garba, 2021) were well tolerated, with no toxic manifestations. Similarly, studies using multiple solvent extracts at high doses reported no significant toxicity (Devaraj et al., 2007). Nevertheless, none of the included studies assessed chronic toxicity, herb–drug interactions, or long-term safety. Given that PUD often requires prolonged management, rigorous toxicological profiling is essential before advancing to clinical trials.

### 4.6 Geographic distribution and ethnopharmacological relevance

Most studies originated from Nigeria and India, reflecting both the availability of *M. oleifera* and its integration into traditional medicine systems in these regions (Devaraj et al., 2007; Alissa et al., 2025; Choudhary et al., 2013; Lawal et al., 2018; Debnath et al., 2011; Abdu and Garba, 2021; Airaodion et al., 2019; Dahiru et al., 2006; Das et al., 2011; Debnath and Guha, 2007; Patel and Lariya, 2019). This cultural relevance underscores the translational importance of our findings, as validating such ethnomedicinal practices could expand affordable therapeutic options in resource-limited settings.

### 4.7 Limitations

Several limitations temper our conclusions. Methodological heterogeneity was evident in ulcer induction methods (ethanol, NSAIDs, pylorus ligation, stress, and acetic acid models) and outcome measures (ulcer index, gastric secretion, histology, biochemical markers). Such variation complicates direct comparisons and likely contributes to the high heterogeneity observed in meta-analyses. Likewise, variations in extraction techniques, ranging from the type of solvent to purification methods, may lead to different concentrations and compositions of bioactive compounds, impacting efficacy. Using different animal strains or species also introduces biological variability, as genetic and physiological differences can influence susceptibility to ulcers and responses to interventions. Publication bias was also detected, with a tendency toward reporting positive results. Furthermore, many studies lacked details on randomization, allocation concealment, or blinding, raising concerns about reproducibility. Importantly, no clinical trials were available, leaving translational gaps unaddressed.

## 5 Conclusion

This systematic review and meta-analysis provide strong preclinical evidence that *Moringa oleifera* possesses gastroprotective and ulcer-healing properties mediated through antioxidant, anti-inflammatory, cytoprotective, and serotonergic pathways. While its activity is consistent across multiple ulcer models, variability in extraction methods, study design, and outcome measures limit the certainty of conclusions. Comparative analyses suggest that *M. oleifera* is promising as an adjunctive therapy but does not consistently match standard drugs.

### 5.1 Recommendations

Based on our findings, we propose several priorities for future research and policy. First, standardized extraction protocols should be established to ensure reproducibility and enable reliable dose–response analyses. Second, comprehensive toxicological studies, including assessments of herb–drug interactions, are essential to address safety concerns. Third, translational research should advance to early-phase clinical trials to evaluate the efficacy, safety, and tolerability of *M. oleifera* in humans to test the efficacy of *Moringa oleifera* in the management of peptic ulcer.

Given its accessibility and affordability, such trials could have a significant impact in resource-limited settings where PUD remains highly prevalent and the cost of conventional drugs is prohibitive. Policymakers are encouraged to support these initiatives as part of broader health strategies aimed at integrating validated ethnopharmacological therapies into healthcare systems. Doing so could reduce the socioeconomic burden of PUD, expand access to safe treatments, and promote culturally aligned healthcare practices.

## Data Availability

The datasets used and/or analysed during the current study are available from the corresponding author on reasonable request.

## References

[B1] Abdu H. Garba A. (2021). Proximate analysis and anti-ulcer activity of methanolic extract of Moringa oleifera. Afr. Scholar J. Agric. Agric. Tech. (JAAT-1) 20 (1), 189–201.

[B2] Ahmed S. R. Rabbee M. F. Roy A. Chowdhury R. Banik A. Kubra K. (2021). Therapeutic promises of medicinal plants in Bangladesh and their bioactive compounds against ulcers and inflammatory diseases. Plants 10 (7), 1348. 10.3390/plants10071348 34371551 PMC8309353

[B3] Airaodion A. I. Olayeri I. M. Ewa A. O. Ogbuagu E. O. Ogbuagu U. Akinmolayan J. D. (2019). Evaluation of *Moringa oleifera* leaf potential in the prevention of peptic ulcer in Wistar rats. Int. J. Res. 6.

[B4] Akhtar N. Syakir Ishak M. I. Bhawani S. A. Umar K. (2021). Various natural and anthropogenic factors responsible for water quality degradation: a review. Water 13 (19), 2660. 10.3390/w13192660

[B5] Alissa M. Bello K. E. Abusalim G. S. (2025). Fraction K3 from *Moringa oleifera* exhibits gastroprotective effects through inhibitory activity against *Helicobacter pylori* and gastric acid secretion. Chem. Biodivers. 22, e00580. 10.1002/cbdv.202500580 40460285

[B6] Bapan B. Ajanta M. Priyanka B. Das Malay K. (2022). Isolation, characterization and evaluation of anti-ulcer activity of phytoconstituents present in *Moringa oleifera* leaf. Int. J. Pharm. Sci. Res. 21, 1218–1224.

[B7] Chaudhary P. Janmeda P. Docea A. O. Yeskaliyeva B. Abdull Razis A. F. Modu B. (2023). Oxidative stress, free radicals and antioxidants: potential crosstalk in the pathophysiology of human diseases. Front. Chem. 11, 1158198. 10.3389/fchem.2023.1158198 37234200 PMC10206224

[B8] Choudhary M. K. Bodakhe S. H. Gupta S. K. (2013). Assessment of the antiulcer potential of *Moringa oleifera* root-bark extract in rats. J. Acupunct. Meridian Stud. 6 (4), 214–220. 10.1016/j.jams.2013.07.003 23972244

[B9] Dahiru D. Onubiyi J. A. Umaru H. A. (2006). Phytochemical screening and antiulcerogenic effect of Moringa oleifera aqueous leaf extract. Afr. J. Traditional Complement. Altern. Med. 3 (3), 70–75. 10.4314/ajtcam.v3i3.31167

[B10] Dalhoumi W. Guesmi F. Bouzidi A. Akermi S. Hfaiedh N. Saidi I. (2022). Therapeutic strategies of *Moringa oleifera* Lam. (Moringaceae) for stomach and forestomach ulceration induced by HCl/EtOH in rat model. Saudi J. Biol. Sci. 29 (6), 103284. 10.1016/j.sjbs.2022.103284 35602868 PMC9118151

[B11] Das D. Dash D. Mandal T. Kishore A. Bairy K. L. (2011). Protective effects of *Moringa oleifera* on experimentally induced gastric ulcers in rats. Res. J. Pharm. Biol. Chem. Sci. 2 (2), 50–55.

[B12] Das A. K. Kalra S. Joshi S. Mithal A. Kumar K. M. P. Unnikrishnan A. G. (2023). The LongitudinAl nationwide stuDy on management and real-world outComes of diabetes in India over 3 years (LANDMARC trial). Endocrinol. Diabetes Metab. 6 (5), e422. 10.1002/edm2.422 37392036 PMC10495555

[B13] Debnath S. Guha D. (2007). Role of *Moringa oleifera* on enterochromaffin cell count and serotonin content of experimental ulcer model. Indian J. Exp. Biol. 45 (8), 726–731. 17877150

[B14] Debnath S. Biswas D. Ray K. Guha D. (2011). *Moringa oleifera* induced potentiation of serotonin release by 5-HT(3) receptors in experimental ulcer model. Phytomedicine 18 (2-3), 91–95. 10.1016/j.phymed.2010.06.003 20637582

[B15] Devaraj V. C. Asad M. Prasad S. (2007). Effect of leaves and fruits of moringa oleifera. on gastric and duodenal ulcers. Pharm. Biol. 45 (4), 332–338. 10.1080/13880200701212924

[B16] Egbe P. A. Umeaku C. N. Iheukwumere I. H. Iheukwumere C. M. Onwuasoanya U. F. Ezenwata I. S. (2025). *Helicobacter pylori* inhibition by medicinal plant extracts: an *in vitro* assessment. IPS J. Drug Discov. Res. Rev. 3 (1), 32–37. 10.54117/ijddrr.v3i1.28

[B17] El Mahdy R. Risha S. Sisi A. Sobhy W. (2020). Potential protective effects of sildenafil and moringa on experimentally-induced gastric ulcer in rats. Egypt. J. Cancer Biomed. Res. 4 (1), 43–55. 10.21608/jcbr.20714.1007

[B18] Fahey J. W. (2017). *Moringa oleifera*: a review of the medicinal potential. Acta Hortic. 1158, 209–224. 10.17660/ActaHortic.2017.1158.25

[B19] Hooijmans C. R. Rovers M. M. de Vries R. B. Leenaars M. Ritskes-Hoitinga M. Langendam M. W. (2014). SYRCLE's risk of bias tool for animal studies. BMC Med. Res. Methodol. 14, 43. 10.1186/1471-2288-14-43 24667063 PMC4230647

[B20] Ibrahim I. A. Al-Qadhi H. I. (2025). The anti ulcerogenic effect of sildenafil and moringa on ulcers in rats. Tissue Cell 93, 102685. 10.1016/j.tice.2024.102685 39765139

[B21] Jo P. Aworunse O. S. Oyesola L. O. Akinnola O. O. Obembe O. O. (2020). A systematic review of pharmacological activities and safety of *Moringa oleifera* . J. Herbmed Pharmacol. 9 (3), 174–190. 10.34172/jhp.2020.24

[B22] Lawal F. Garba K. Shuai’bu A. B. Chedi B. A. Z. (2018). Evaluation of the antiulcer activity of aqueous seed extract of Moringa oleifera lamarck (Moringaceae). Trop. J. Nat. Prod. Res. TJNPR. 2 (3), 140–144. 10.26538/tjnpr/v2i3.8

[B23] Mabrok H. Mohamed M. (2019). Induction of COX-1, suppression of COX-2 and pro-inflammatory cytokines gene expression by moringa leaves and its aqueous extract in aspirin-induced gastric ulcer rats. Mol. Biol. Rep. 46 (4), 4213–4224. 10.1007/s11033-019-04874-9 31111367

[B24] Malfertheiner P. Chan F. K. McColl K. E. (2009). Peptic ulcer disease. Lancet 374 (9699), 1449–1461. 10.1016/S0140-6736(09)60938-7 19683340

[B25] Olajide K. A. Olukayode E. V. Sina I. Avwioro G. Anderson L. E. (2024). Synergistic nutraceutical effects of aqueous extracts from Vernonia amygdalina and *Moringa oleifera*: a promising therapeutic strategy for gastric ulcer management. J. Prev. Diagnostic Treat. Strategies Med. 3 (4), 296–302. 10.4103/jpdtsm.jpdtsm_102_24

[B26] Page M. J. McKenzie J. E. Bossuyt P. M. Boutron I. Hoffmann T. C. Mulrow C. D. (2021). The PRISMA 2020 statement: an updated guideline for reporting systematic reviews. Syst. Rev. 10 (1), 89. 10.1186/s13643-021-01626-4 33781348 PMC8008539

[B27] Pareek A. Pant M. Gupta M. M. Kashania P. Ratan Y. Jain V. (2023). *Moringa oleifera*: an updated comprehensive review of its pharmacological activities, ethnomedicinal, phytopharmaceutical formulation, clinical, phytochemical, and toxicological aspects. Int. J. Mol. Sci. 24 (3), 2098. 10.3390/ijms24032098 36768420 PMC9916933

[B28] Patel V. K. Lariya N. K. (2019). Anti-ulcer activity of extract of *Moringa oleifera* Lam. using pylorus ligation induced ulce. Pharma Innov. J. 8 (6), 247–250.

[B29] Patel R. Montagut-Bordas C. Dickenson A. H. (2018). Calcium channel modulation as a target in chronic pain control. Br. J. Pharmacol. 175 (12), 2173–2184. 10.1111/bph.13789 28320042 PMC5980588

[B30] Pollock A. Farmer S. E. Brady M. C. Langhorne P. Mead G. E. Mehrholz J. (2016). An algorithm was developed to assign GRADE levels of evidence to comparisons within systematic reviews. J. Clin. Epidemiol. 70, 106–110. 10.1016/j.jclinepi.2015.08.013 26341023 PMC4742519

[B31] Ruckmani K. Kavimani S. Jayakar B. Anandan R. (1998). Anti-ulcer activity of the alkali preparation of the root and fresh leaf juice of *Moringa oleifera* lam. Anc. Sci. Life 17 (3), 220–223. 22556845 PMC3331106

[B32] Soto J. A. Gómez A. C. Vásquez M. Barreto A. N. Molina K. S. Zuniga-Gonzalez C. A. (2025). Biological properties of *Moringa oleifera*: a systematic review of the last decade. F1000Res 13, 1390. 10.12688/f1000research.157194.2 39895949 PMC11782934

[B33] Serafim C. Araruna M. E. Júnior E. A. Diniz M. Hiruma-Lima C. Batista L. (2020). A review of the role of flavonoids in peptic ulcer (2010-2020). Molecules 25 (22), 5431. 10.3390/molecules25225431 33233494 PMC7699562

[B34] van Gestel L. C. Adriaanse M. A. Kanis S. L. Mensink-Bout S. M. Schoones J. W. Numans M. E. (2024). Determinants of and interventions for proton pump inhibitor prescription behavior: a systematic scoping review. BMC Prim. Care 25, 208. 10.1186/s12875-024-02459-5 38862886 PMC11165893

[B35] Wahba H. M. A. Shelbaya L. A. (2018). Effects of *Moringa oleifera* L. Herb and its extract on indomethacin-induced gastric oxidative stress in rats. Biosci. Res. 15 (3), 1917–1924.

[B36] Wang D. Chen H. Luo Y. (2024). Histamine H2 receptor antagonists in the treatment and prevention of heart failure. Int. J. Gen. Med. 17, 6047–6052. 10.2147/IJGM.S499182 39678682 PMC11646443

