# Supplementary material for: Preclinical evidence of the therapeutic effect of Moringa oleifera in peptic ulcer disease: a systematic review and meta-analysis
Source: Front Pharmacol. 2026 Mar 20;17:1689789. doi: 10.3389/fphar.2026.1689789 (PMC13047084; doi:10.3389/fphar.2026.1689789)
Supplement: Supplementary file 2 [file Supplementaryfile5.docx]

**Supplementary Table 5: SYRCLE’S RoB for the therapeutic effect of *Moringa oleifera* in the treatment of peptic ulcer disease: A systematic review and meta-analysis**

| **S/N** | **Author's**  **/Year** | **Was the sample frame appropriate for addressing the target animal population?** | **Were animals (study participants) allocated in an appropriate way?** | **Was the sample size adequate?** | **Were the study animals and experimental setting described in detail?** | **Was the data analysis conducted with sufficient coverage of the identified sample?** | **Were valid and reliable methods used for the induction and assessment of peptic ulcers?** | **Was the condition measured in a standard, reliable way for all participants?** | **Was there appropriate statisticalanalysis?** | **Was the outcome (ulcer healing, severity, etc.) measured in a standard and reliable way for all animals?** | **Total score** | **Grade** |
| --- | --- | --- | --- | --- | --- | --- | --- | --- | --- | --- | --- | --- |
| 1 | Devaraj et al. (2007) | 1 | 1 | 1 | 1 | 1 | 1 | 1 | 1 | 1 | 9 | Low |
| 2 | Dahiru et al. (2006) | 1 | 0 | 1 | 1 | 1 | 1 | 1 | 1 | 1 | 8 | Low |
| 3 | Alissa et al. (2025) | 1 | 1 | 1 | 1 | 1 | 1 | 1 | 1 | 1 | 9 | Low |
| 4 | Lawal et al. (2018) | 1 | 1 | 1 | 1 | 1 | 1 | 1 | 1 | 1 | 9 | Low |
| 5 | Das et al. (2011) | 0 | 0 | 0 | 1 | 1 | 1 | 1 | 1 | 1 | 6 | Mid |
| 6 | Choudhary et al. (2013) | 1 | 0 | 1 | 1 | 1 | 1 | 1 | 1 | 1 | 8 | Low |
| 7 | Debnath & Guha (2007) | 1 | 1 | 1 | 1 | 1 | 1 | 1 | 0 | 1 | 8 | Low |
| 8 | Debnath et al. (2011) | 1 | 1 | 1 | 1 | 1 | 1 | 1 | 1 | 1 | 9 | Low |
| 9 | Patel & Lariya (2019) | 1 | 1 | 1 | 1 | 1 | 1 | 1 | 1 | 1 | 9 | Low |
| 10 | Hamisu & Ashiru, 2021) | 0 | 0 | 1 | 1 | 1 | 1 | 1 | 1 | 1 | 7 | Mid |
| 11 | Airaodion et al., (2019) | 1 | 0 | 1 | 1 | 1 | 1 | 0 | 0 | 1 | 6 | Mid |
