# Supplementary material for: Preclinical evidence of the therapeutic effect of Moringa oleifera in peptic ulcer disease: a systematic review and meta-analysis
Source: Front Pharmacol. 2026 Mar 20;17:1689789. doi: 10.3389/fphar.2026.1689789 (PMC13047084; doi:10.3389/fphar.2026.1689789)
Supplement: Supplementary file 3 [file Supplementaryfile2.docx]

**Supplementary Table 2a: PUBMED SEARCH**

| **S/No** | **Keywords** | **Search terms** | **Results** |
| --- | --- | --- | --- |
| #1 | *Moringa oleifera* | ((((((("M. oleifera"[Title/Abstract]) OR ("horseradish tree"[Title/Abstract])) OR ("ben oil tree"[Title/Abstract])) OR ("miracle tree"[Title/Abstract])) OR ("drumstick tree"[Title/Abstract])) OR (moringa[Title/Abstract])) OR ("moringa oleifera extract"[Title/Abstract])) OR ("Moringa oleifera"[Title/Abstract]) | [2,823](https://pubmed.ncbi.nlm.nih.gov/?term=%28%28%28%28%28%28%28%22M.+oleifera%22%5BTitle%2FAbstract%5D%29+OR+%28%22horseradish+tree%22%5BTitle%2FAbstract%5D%29%29+OR+%28%22ben+oil+tree%22%5BTitle%2FAbstract%5D%29%29+OR+%28%22miracle+tree%22%5BTitle%2FAbstract%5D%29%29+OR+%28%22drumstick+tree%22%5BTitle%2FAbstract%5D%29%29+OR+%28moringa%5BTitle%2FAbstract%5D%29%29+OR+%28%22moringa+oleifera+extract%22%5BTitle%2FAbstract%5D%29%29+OR+%28%22Moringa+oleifera%22%5BTitle%2FAbstract%5D%29&ac=no&sort=relevance) |
| #2 | peptic ulcer disease | (((((((((("Peptic ulcer disease*"[Title/Abstract]) OR ("Gastroduodenal ulcer*"[Title/Abstract])) OR (ulcer*[Title/Abstract])) OR ("duodenal ulcer*"[Title/Abstract])) OR ("helicobacter pylori"[Title/Abstract])) OR ("ulcer disease*"[Title/Abstract])) OR ("gastric ulcer*"[Title/Abstract])) OR ("peptic ulcer*"[Title/Abstract])) OR ("stomach ulcer*"[Title/Abstract])) OR ("H. pylori"[Title/Abstract])) OR (PUD[Title/Abstract]) | [299,943](https://pubmed.ncbi.nlm.nih.gov/?term=%28%28%28%28%28%28%28%28%28%28%22Peptic+ulcer+disease%2A%22%5BTitle%2FAbstract%5D%29+OR+%28%22Gastroduodenal+ulcer%2A%22%5BTitle%2FAbstract%5D%29%29+OR+%28ulcer%2A%5BTitle%2FAbstract%5D%29%29+OR+%28%22duodenal+ulcer%2A%22%5BTitle%2FAbstract%5D%29%29+OR+%28%22helicobacter+pylori%22%5BTitle%2FAbstract%5D%29%29+OR+%28%22ulcer+disease%2A%22%5BTitle%2FAbstract%5D%29%29+OR+%28%22gastric+ulcer%2A%22%5BTitle%2FAbstract%5D%29%29+OR+%28%22peptic+ulcer%2A%22%5BTitle%2FAbstract%5D%29%29+OR+%28%22stomach+ulcer%2A%22%5BTitle%2FAbstract%5D%29%29+OR+%28%22H.+pylori%22%5BTitle%2FAbstract%5D%29%29+OR+%28PUD%5BTitle%2FAbstract%5D%29&ac=no&sort=relevance) |
| #3 |  | #1 AND #2 | [46](https://pubmed.ncbi.nlm.nih.gov/?term=%231+AND+%232&ac=no&sort=relevance) |

**Supplementary Table 2b: WEB OF SCIENCE**

| **S/No** | **Keywords** | **Search terms** | **Results** |
| --- | --- | --- | --- |
| #1 | *Moringa oleifera* | TS=("M. oleifera" OR "horseradish tree" OR "ben oil tree" OR "miracle tree" OR "drumstick tree" OR moringa OR "moringa oleifera extract" OR "Moringa oleifera") | [7,641](https://www.webofscience.com/wos/woscc/summary/fa7bdeec-e95d-4f46-b30e-da716511f89d-016b2127b9/relevance/1) |
| #2 | peptic ulcer disease | TS=("Peptic ulcer disease*" OR "Gastroduodenal ulcer*" OR ulcer* OR "duodenal ulcer*" OR "helicobacter pylori" OR "ulcer disease*" OR "gastric ulcer*" OR "peptic ulcer*" OR "stomach ulcer*" OR "H. pylori" OR PUD) | [303,670](https://www.webofscience.com/wos/woscc/summary/8cbad9ac-87c2-4e67-a00d-a26bd3d08870-016b213769/relevance/1) |
| #3 |  | #2 AND #1 | [74](https://www.webofscience.com/wos/woscc/summary/cb92b73a-8ea9-4ee6-b70f-466a1f21c5cd-016b213897/relevance/1) |

**Supplementary Table 2c: CINAHL Search**

| **S/No** | **Keywords** | **Search terms** | **Results** |
| --- | --- | --- | --- |
| #1 |  | XB ("M. oleifera" OR "horseradish tree" OR "ben oil tree" OR "miracle tree" OR "drumstick tree" OR moringa OR "moringa oleifera extract" OR "Moringa oleifera") AND XB ("Peptic ulcer disease*" OR "Gastroduodenal ulcer*" OR ulcer* OR "duodenal ulcer*" OR "helicobacter pylori" OR "ulcer disease*" OR "gastric ulcer*" OR "peptic ulcer*" OR "stomach ulcer*" OR "H. pylori" OR PUD) | 7 |

**Supplementary Table 2d: SCOPUS search**

| **S/No** | **Keywords** | **Search terms** | **Results** |
| --- | --- | --- | --- |
| #1 | *M. oleifera* | "M. oleifera" OR "horseradish tree" OR "ben oil tree" OR "miracle tree" OR "drumstick tree" OR moringa OR "moringa oleifera extract" OR "Moringa oleifera"  AND  "Peptic ulcer disease*" OR "Gastroduodenal ulcer*" OR ulcer* OR "duodenal ulcer*" OR "helicobacter pylori" OR "ulcer disease*" OR "gastric ulcer*" OR "peptic ulcer*" OR "stomach ulcer*" OR "H. pylori" OR PUD | 141 |
